# Predictive biomarkers of lithium response: preliminary results from the European R-LiNK initiative

**DOI:** 10.1192/j.eurpsy.2026.10395

**Published:** 2026-07-31

**Authors:** F. Bellivier, R-LiNK Consortium members

**Affiliations:** Therapeutic Optimization in Neuropharmacology, Université Paris Cité, INSERM UMRS 1144, Paris, France; Department of Psychiatry and Addiction Medicine, Hôpitaux Lariboisière-Fernand Widal, DMU Neurosciences, AP-HP.Nord, Université Paris Cité, Paris, France

## Abstract

**Abstract:**

Lithium (Li) remains the gold standard for long-term bipolar disorder management, significantly reducing relapse, hospitalisation, suicidal behaviour, and all-cause mortality (Cipriani et al., 2013; Geddes et al., 2004; Pompili et al., 2025). However, only one-third of patients achieve an excellent response, while others experience partial or no benefit (Alda, 2015). The identification of markers predicting Li treatment response is crucial to optimise benefit-risk ratios, guide personalised treatment strategies and avoid empirical lengthy trials.

We present innovative longitudinal study designs and breakthroughs in -omics and neuroimaging, with a focus on the preliminary results of EU Horizon 2020-funded R-LiNK study. Our final goal is to translate these findings into clinical practice, reducing trial-and-error prescribing and improving patient outcomes.

The R-LiNK consortium conducted a landmark multinational, multicentre study to identify biomarkers of lithium response and tolerability (Scott et al., 2019). A total of 169 patients initiating lithium were followed for 24 months, with biomarker assessments at baseline and 3 months. Response was evaluated monthly using the Alda scale and expert consensus.

**Key highlights include:**

- **Study design and challenges:** Overview of R-LiNK’s approach, addressing methodological hurdles in defining lithium response and tolerability in real-world settings.
- **Preliminary results** include (i) significant reduction of suicide risk 3 months after Li initiation, (ii) baseline Gray Matter (GM) in hippocampus and amygdala associated with Li response, (iii) global GM volume increase between baseline and Month 3, (iv) GM volume increase between baseline and Month 3 in hippocampus and amygdala, (v) using Li-MRI, lower Li signal in several brain regions associated with good response and (vi) Li-induced molecular signature in blood was different in males and females.
- **Neuroimaging innovations:** Harmonised MRI (structural, diffusion-weighted, proton spectroscopy) and pioneering multinuclear 7Li-MRI—implemented in six centres—to map lithium’s brain distribution. Findings from individual modalities and plans for multimodal data fusion will be discussed.

**Attendees will leave with:**

- A comprehensive update on research efforts aiming at the identification of Li response biomarkers and their clinical implication.
- Practical implications of integrating advanced technologies into psychiatric care in the future.

**References:**

Alda, M. (2015). *Molecular Psychiatry, 20*(6), 661-670. Cipriani, A., et al. (2013). *BMJ, 346*, f3646. Geddes, J. R., et al. (2004). *American Journal of Psychiatry, 161*(2), 217-222. Pompili, M., et al. (2025). *Acta Psychiatrica Scandinavica, 152*(4), 290-298. Scott, J., et al. (2019). *International Journal of Bipolar Disorders, 7*
(1), 20.

**Disclosure of Interest:**

None Declared

